# Study of Telomere Length Kinetics and Telomerase Reverse Transcriptase Gene Polymorphism in Children Undergoing Allogeneic Haematopoietic Stem Cell Transplantation

**DOI:** 10.3390/genes17091050

**Published:** 2026-08-30

**Authors:** Katharina Häusler, Marta Dratwa-Kuzmin, Marek Ussowicz, Blanka Rybka, Renata Ryczan-Krawczyk, Krzysztof Kalwak, Katarzyna Bogunia-Kubik

**Affiliations:** 1 Laboratory of Clinical Immunogenetics and Pharmacogenetics, Hirszfeld Institute of Immunology and Experimental Therapy, Polish Academy of Sciences, 53-114 Wrocław, Poland; katharina.hausler@umw.edu.pl; 2 Department of Toxicology, Faculty of Pharmacy, Wroclaw Medical University, 50-556 Wrocław, Poland; 3 Department and Clinic of Paediatric Bone Marrow Transplantation, Oncology and Haematology, Wroclaw Medical University, 50-556 Wrocław, Poland; marek.ussowicz@umw.edu.pl (M.U.); blanka.rybka@umw.edu.pl (B.R.); renata.ryczan@umw.edu.pl (R.R.-K.); krzysztof.kalwak@umw.edu.pl (K.K.)

**Keywords:** allogeneic haematopoietic stem cell transplantation in children, telomere length, *TERT* gene polymorphism, graft-versus-host disease, chimerism, infections, survival

## Abstract

**Background:** Telomere length and telomerase activity are important determinants of haematopoietic cell proliferative capacity and may influence outcomes after allogeneic haematopoietic stem cell transplantation (allo-HSCT). Genetic variation within the telomerase reverse transcriptase (*TERT*) gene may further contribute to transplantation success and post-transplant complications. **Methods:** Telomere length was assessed in 98 paediatric allo-HSCT recipients and their donors using quantitative real-time PCR before allo-HSCT and at one and two years post-transplantation. Selected four *TERT* polymorphisms (rs2736100, rs2853669, rs2735940, and rs10069690) were genotyped in donors and recipients before allo-HSCT. Associations between telomere length, genetic variants, and clinical outcomes were analysed. **Results:** Telomere length did not differ between recipients and donors before transplantation; however, donors younger than 18 years exhibited significantly longer telomeres. Telomere length increased at one and two years after allo-HSCT compared with pre-transplant values, with the most pronounced changes observed in patients with acute lymphoblastic leukaemia. No direct associations were found between *TERT* polymorphisms and telomere length. Nevertheless, the recipient rs2735940 *T* allele was associated with improved survival, the donor rs2736100 *G* allele with the achievement of complete chimerism, and the recipient rs2853669 *C* allele with an increased risk of acute graft-versus-host disease (aGvHD). Haplotype analysis identified associations between *TERT* variants and cytomegalovirus reactivation as well as aGvHD. **Conclusions:** Paediatric allo-HSCT is associated with dynamic post-transplant changes in telomere length. Although *TERT* polymorphisms did not directly affect telomere length, several variants were linked with clinically relevant transplantation outcomes, highlighting the potential role of telomere biology in haematopoietic reconstitution and transplant-related complications.

## 1. Introduction

Haematopoietic stem cell transplantation (HSCT) is a known therapeutic procedure used in the treatment of haematopoietic proliferative disorders as well as some non-malignant diseases, including congenital and metabolic disorders [1]. The procedure involves the transplantation of hematopoietic stem cells from a suitable donor to restore normal haematopoiesis in the recipient [2]. In allogeneic HSCT (allo-HSCT), the optimal donor is typically a human leukocyte antigen (HLA)-matched sibling (MSD), while in the absence of such a donor, a matched unrelated donor (MUD) may be used. Allo-HSCT remains the standard of care for many haematological malignancies, including acute lymphoblastic leukaemia (ALL) [3,4,5].

The outcome of allo-HSCT depends on multiple recipient- and donor-related factors. Recipient-associated factors include disease status, age, prior treatment response, conditioning regimens, stem cell source, and infections, while donor-related factors are age, sex, infectious status, and HLA compatibility. Following transplantation, hematopoietic and immune reconstitution is initiated by engraftment, typically occurring within 10–21 days and confirmed by donor-derived haematopoiesis using chimerism analysis [6,7]. The success of HSCT is measured at the stage of chimerism 100 days after the transplant, where full chimerism is the goal.

The need for rapid proliferation of donor-derived cells during hematopoietic recovery links HSCT success with telomere biology. Telomeres are DNA–protein structures located at chromosome ends that maintain genomic stability [8,9]. With each cell division, telomeres progressively shorten, although telomerase activity can partially counteract this process [8,10]. Telomere dysfunction leads to genomic instability and cellular senescence, which is particularly relevant in the context of intensive post-transplant proliferation [9,10,11]. Accordingly, telomere length has emerged as an important determinant of HSCT outcomes. Shorter, age-adjusted recipient telomere length is associated with higher transplant-related mortality (TRM) and increased early non-relapse mortality, especially after myeloablative conditioning [12,13].

These observations suggest that, next to HLA matching, donor selection may also be influenced by biological features, such as the telomere length of the donor and recipient. It is shown that transplant recipients exhibit shorter telomeres compared with donors, and factors such as donor characteristics and post-transplant complications may contribute to accelerated telomere shortening [14,15].

Despite its therapeutic potential, HSCT is associated with significant complications that remain a major cause of morbidity and mortality. Infectious complications are predominant, with cytomegalovirus (CMV) reactivation representing a key clinical challenge [16]. CMV persists in 40–80% of the population and often reactivates under immunosuppression, particularly in the context of allogeneic transplantation, prior seropositivity, advanced age, and acute graft-versus-host disease (aGvHD) [17]. Acute GvHD is another major complication, usually occurring within 100 days after transplantation and affecting approximately 35–50% of recipients [18,19]. It is an immune-mediated process driven by donor-derived T lymphocytes and primarily involves the skin, liver, and gastrointestinal tract [20,21]. Its severity depends on multiple factors, including conditioning intensity, stem cell source, patient age, and donor characteristics, particularly in the case of unrelated or mismatched donors [19,22,23].

Importantly, both infectious complications and aGvHD may increase proliferative stress on haematopoietic cells, thereby influencing telomere dynamics and long-term transplant outcomes. In this context, genetic variation within telomere-related pathways, particularly within the telomerase reverse transcriptase (*TERT*), represents an additional factor modulating HSCT success. The *TERT* gene encodes the catalytic subunit of telomerase and plays a key role in telomere maintenance [24,25].

Single nucleotide polymorphisms (SNPs) within the *TERT* locus have been associated with cancer susceptibility as well as HSCT outcomes [26]. Variants such as rs2736100, rs10069690, rs2735940, and rs2853669 influence telomerase activity and gene expression and have been linked to acute lymphoblastic leukaemia risk and clinical outcomes [27]. Moreover, selected TERT polymorphisms have been associated with overall mortality and increased risk of aGvHD in HSCT recipients [28,29,30].

The goal of this study was to evaluate changes in mean telomere length in paediatric patients undergoing allogeneic HSCT at three time points: before and prospectively at one and two years after transplantation. Additionally, donor telomere length was assessed as a potential factor influencing transplant outcomes. Furthermore, the study investigated the impact of polymorphic variation within the *TERT* gene (rs2736100, rs2853669, rs2735940, and rs10069690), analysed in both donors and recipients, on transplantation success and the occurrence of post-transplant complications.

## 2. Materials and Methods

### 2.1. Patients and Donors

All children were treated at the Department and Clinic of Paediatric Bone Marrow Transplantation, Oncology, and Haematology, Wroclaw Medical University in Poland. The study was approved by the Wroclaw Medical University Ethics Committee (identification code KB-561/2019). The research material consisted of peripheral blood collected from patients and their donors before transplantation (98 donor–recipient pairs) as well as from patients one year (*n* = 78) and two years (*n* = 75) after allo-HSCT. The patients characteristics and transplant details are presented in Table 1.

### 2.2. Genomic DNA Extraction

Genomic DNA was isolated from 200 μL of EDTA-anticoagulated peripheral whole blood using the NucleoSpin^®^ Blood kit (Macherey-Nagel, Düren, Germany) according to the manufacturer’s instructions. DNA was eluted in 100 μL of pre-warmed (70 °C) elution buffer BE and stored at −25 °C until further analyses. DNA concentration and purity were assessed spectrophotometrically using a NanoDrop DeNovix DS-11 spectrophotometer (DeNovix Inc., Wilmington, DE, USA).

### 2.3. Mean Telomere Length Analysis

Mean telomere length was determined using the Absolute Human Telomere Length Quantification qPCR Assay Kit (ScienCell, Carlsbad, CA, USA, Cat. No. 8918) according to the manufacturer’s protocol. Quantitative real-time PCR (qPCR) reactions were performed on a LightCycler^®^ 480 II Real-Time PCR System (Roche Diagnostics International, Rotkreuz, Switzerland). For each sample, telomeric repeats and a single-copy reference (SCR) gene located on chromosome 17 were amplified in separate reactions. Each reaction was performed in duplicate, and the mean Cq value was used for further calculations. A reference genomic DNA sample with a known telomere length, provided by the manufacturer, was included in each run as a positive control. Absolute telomere lengths were calculated using the ΔΔCq method, following the manufacturer’s guidelines.

### 2.4. Genotyping of TERT Gene Polymorphisms

Genotyping of *TERT* gene polymorphisms (rs10069690, rs2735940, rs2736100, and rs2853669) was performed using LightSNiP typing assays (TIB MOLBIOL, Berlin-Bezirk Tempelhof-Schöneberg, Germany). Real-time PCR amplification was carried out on a LightCycler^®^ 480 II system (Roche Diagnostics International, Rotkreuz, Switzerland). Cycling conditions included an initial denaturation at 95 °C for 10 min, followed by 45 cycles of denaturation, annealing, and elongation. Following PCR amplification, melting curve analysis was conducted to identify allelic variants of the analysed polymorphisms.

### 2.5. Statistical Analysis

Statistical analyses were performed using GraphPad Prism 8, the R statistical environment, and a web-based application developed in Streamlit using a custom Python 3.12 script. Data distribution was assessed using the Shapiro–Wilk test. Depending on data normality, parametric tests (unpaired Student’s *t*-test, one-way ANOVA) or non-parametric tests (Mann–Whitney U test, Kruskal–Wallis test) were applied.

Post hoc analyses were conducted using Tukey’s test or Dunn’s test with false discovery rate (FDR) correction according to the Benjamini–Krieger–Yekutieli procedure. Association analyses included calculation of odds ratios (ORs) with 95% confidence intervals (CIs) and evaluation of Hardy–Weinberg equilibrium using the χ^2^ test.

To account for repeated measurements and missing observations, longitudinal changes in telomere length were analysed using a linear mixed-effects model. Time (before transplantation, 1 year, and 2 years after transplantation) was included as a fixed effect, and participant ID was included as a random intercept to account for within-subject correlation. Pairwise comparisons between time points were performed using estimated marginal means with Tukey’s test adjustment for multiple comparisons. Statistical analyses were conducted in R using the lmerTest and emmeans packages.

Linkage disequilibrium (LD) between analysed loci was assessed in R using appropriate packages. Multivariable linear regression models were applied to evaluate the independent effects of clinical factors on telomere length at different time points (before allo-HSCT, one year and two years post-transplantation, and in donors). All tests were two-sided, and *p* values < 0.05 were considered statistically significant.

## 3. Results

### 3.1. Telomere Length in HSCT Recipients and Their Donors Before Transplantation—Relationships with Age, Diagnosis, and Pretransplant Conditioning Regimen

**Figure 1:**
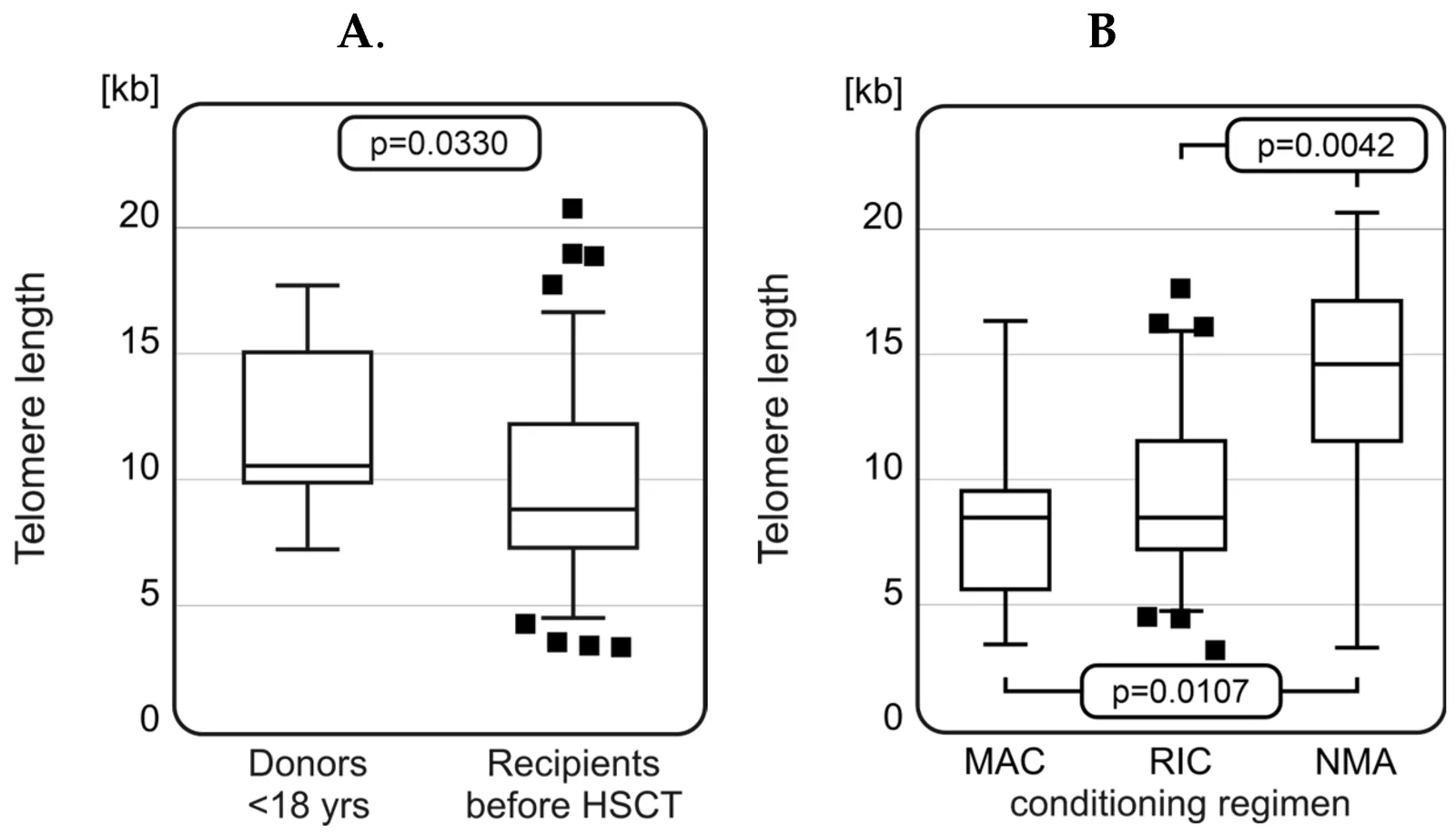
Telomere length before transplantation. Telomere length in paediatric patients and donors below 18 years of age prior to transplantation (**A**) and in recipients who underwent various conditioning regimens (MAC, RIC, NMA) (**B**). The y-axis represents telomere length expressed in kilobases (kb). Data are presented as box-and-whisker plots showing the median (horizontal line), interquartile range (box), and 5th–95th percentile range (whiskers). Individual points indicate individual measurements outside the 5th–95th percentile range. Statistical significance was assessed using (**A**) the Mann–Whitney U test and (**B**) the Kruskal–Wallis test.

A comparative analysis of mean telomere length was performed between donors (10.62 ± 3.85 kb; *n* = 98) and recipients before transplantation (9.97 ± 3.82 kb). No statistically significant difference in telomere length was observed between donors and recipients in the overall analysis. Recipients were stratified into two groups according to the median age of 7 years: younger recipients (≤7 years, *n* = 48) and older recipients (>7 years, *n* = 50). Although no statistically significant difference in telomere length was observed between the two groups, a trend toward longer telomeres was noted in younger recipients (*p* = 0.0597). Specifically, the mean telomere length was greater in younger recipients than in older recipients (10.72 ± 3.84 kb vs. 9.24 ± 3.60 kb, respectively). Given the substantial age heterogeneity within the donor group (median age: 26 years; age range: 3–57 years; Table 1), an additional age-restricted analysis was conducted to better match donor age to recipient age. Donors younger than 18 years were included in this sub-analysis *(n* = 17). This age-adjusted analysis revealed a statistically significant difference in telomere length, with younger donors (<18 years) exhibiting longer telomeres (11.88 ± 3.37 kb) compared with recipients before transplantation (9.97 ± 3.82 kb; *p* = 0.0365) (Figure 1A).

Telomere length in recipients before transplantation was compared across different conditioning regimens classified as myeloablative (MAC), reduced-intensity (RIC), or non-myeloablative (NMA). The analysis revealed a statistically significant difference in telomere length among the conditioning groups (Kruskal–Wallis, *p* = 0.0038).

Post hoc analysis using Dunn’s test showed that recipients in the NMA group (*n* = 13; 13.75 ± 4.86 kb) had significantly longer telomeres compared with patients in the MAC group (*n* = 18; 8.50 ± 3.53 kb; *p* = 0.0042) and the RIC group (*n* = 69; 9.62 ± 3.20 kb; *p* = 0.0107). No significant differences were observed between the MAC and RIC groups (*p* = 0.8879) (Figure 1B). Importantly, the NMA group (*n* = 13) comprised patients with bone marrow failure disorders, including severe/very severe aplastic anaemia, aplastic anaemia, Fanconi anaemia, and paroxysmal nocturnal haemoglobinuria, whereas the MAC group consisted predominantly of patients with ALL. The RIC group was more heterogeneous, comprising both malignant and non-malignant disorders. The distribution of underlying diseases therefore differed substantially across conditioning groups, indicating that conditioning intensity was not independent of clinical characteristics.

### 3.2. Telomere Length Kinetics in Children Undergoing HSCT

**Figure 2:**
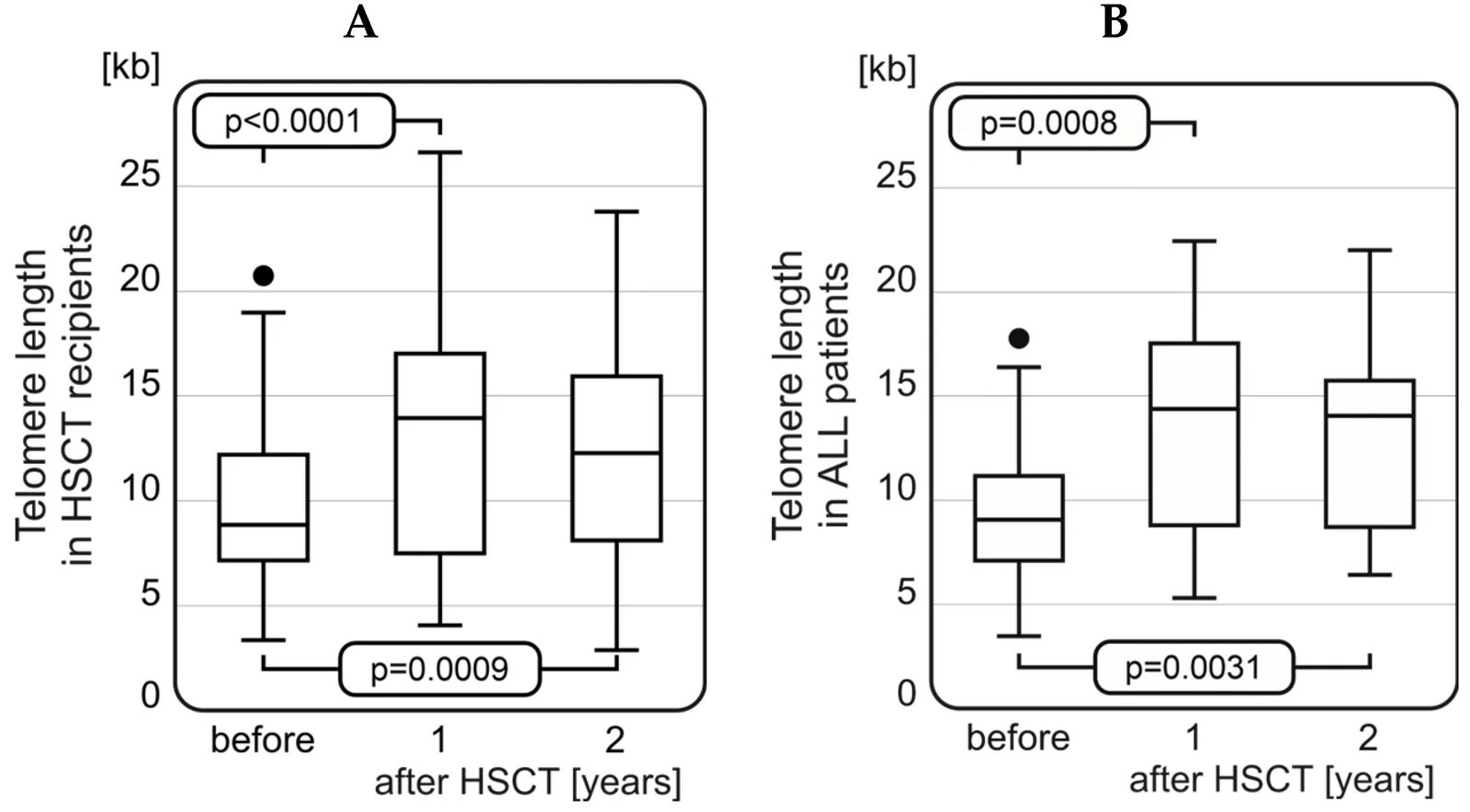
Telomere length kinetics. (**A**) Comparison of telomere length in allo-HSCT recipients before transplantation, one year after transplantation, and two years after transplantation. (**B**) Comparison of telomere length in recipients diagnosed with acute lymphoblastic leukaemia (ALL) at the same three time points. Differences in telomere length over time were analysed using linear mixed-effects models with post hoc pairwise comparisons. The y-axis represents telomere length expressed in kilobases (kb), while the x-axis indicates the three assessment time points. Data are presented as box-and-whisker plots showing the median (horizontal line), interquartile range (box), and 5th–95th percentile range (whiskers). Individual points represent observations outside the 5th–95th percentile range.

Next, we compared telomere length in patients before transplantation and at one and two years after allo-HSCT using a linear mixed-effects model with a random intercept for each patient. A statistically significant difference in telomere length was observed across the analysed time points (Figure 2A). Estimated marginal means demonstrated an increase in telomere length after transplantation, with the highest values observed one year after transplantation (12.67 kb; 95% CI: 11.63–13.7), compared with values before transplantation (9.97 kb; 95% CI: 9.03–10.9). Post hoc analysis with Tukey adjustment revealed significantly longer telomeres at one year after transplantation compared with baseline (before transplantation; *p* < 0.0001) and at two years after transplantation compared with baseline (*p* = 0.0009). No significant difference was observed between one and two years after transplantation (*p* = 0.8625).

To investigate the association between telomere length and the underlying malignancy, telomere dynamics were analysed separately across different disease groups. Among all evaluated malignancies, statistically significant differences were observed only in patients with acute lymphoblastic leukaemia (ALL) (Figure 2B). In this group, estimated marginal means indicated an increase in telomere length after transplantation. Telomere length increased from 9.42 kb (95% CI: 8.05–10.8) before HSCT to 13.04 kb (95% CI: 11.43–14.7) one year after transplantation and remained elevated at two years (12.74 kb; 95% CI: 11.07–14.4). Post hoc analysis demonstrated significant differences between baseline and one year after transplantation (*p* = 0.0008) and between baseline and two years after transplantation (*p* = 0.0031). No significant difference was observed between one and two years after transplantation (*p* = 0.9534).

**Figure 3:**
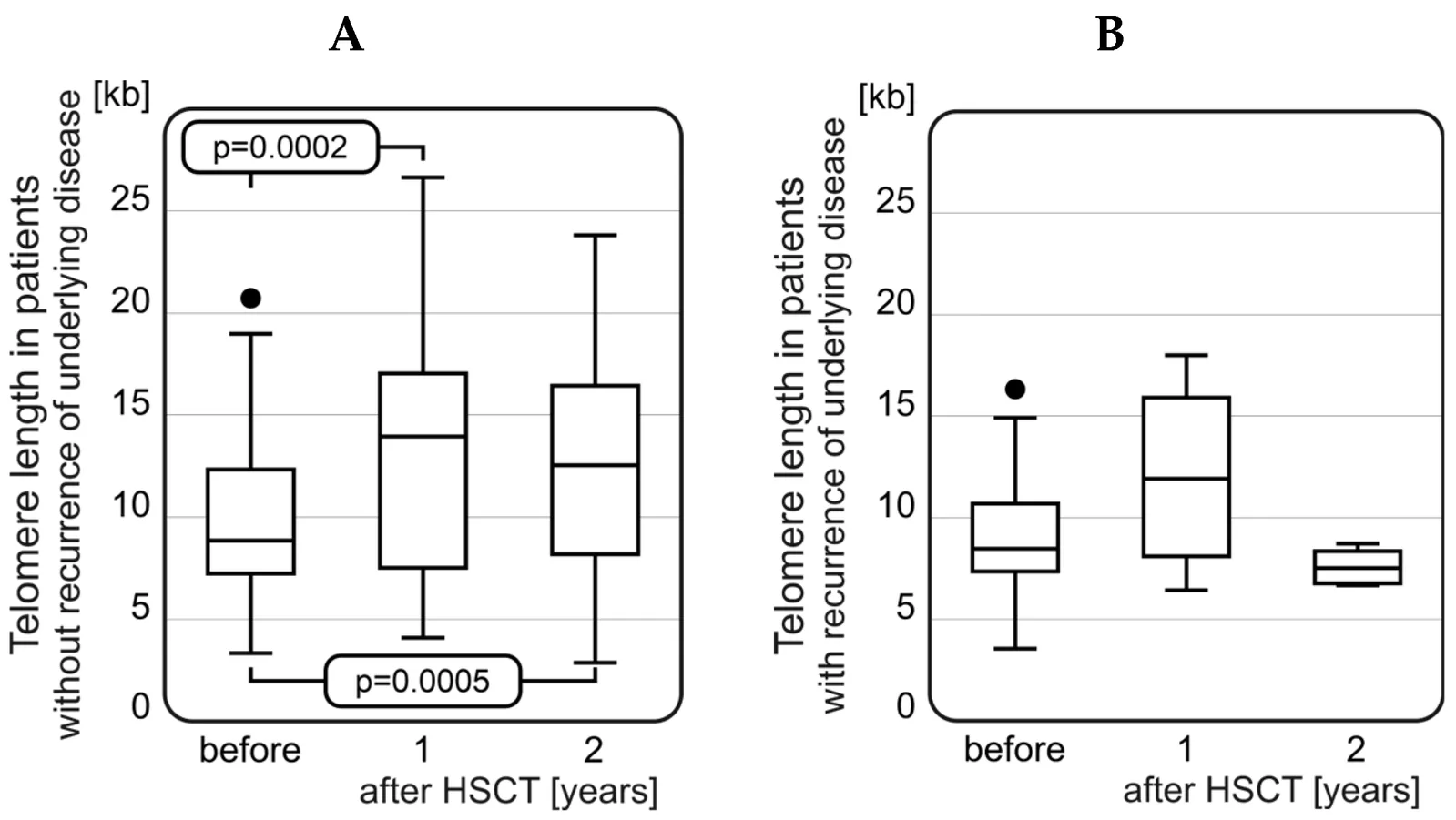
Changes in telomere length before transplantation and at 1- and 2-year follow-up after allo-HSCT depending on recurrence of the underlying disease. (**A**,**B**) Changes in telomere length over time were analysed using linear mixed-effects models with post hoc pairwise comparisons. Data are presented as box-and-whisker plots showing the median (horizontal line), interquartile range (box), and 5th–95th percentile range (whiskers). Individual points represent observations outside the 5th–95th percentile range.

The further analyses evaluated telomere length in HSCT recipients according to the occurrence of post-transplant complications and disease recurrence. Telomere length was assessed at three time points: before transplantation, one year after allo-HSCT, and two years after allo-HSCT (Figure 3). An increase in telomere length over time was observed primarily in patients without recurrence of the underlying disease (Figure 3A). However, these relationships were not observed in patients having and lacking aGvHD or CMV infection.

When patients were categorised according to recurrence of the underlying disease, recipients without recurrence demonstrated significant changes in telomere length over time. Telomere length increased from 10.10 kb (95% CI: 9.06–11.10) before transplantation to 12.70 kb (95% CI: 11.66–13.80) one year after allo-HSCT (*p* = 0.0002) and remained significantly higher at two years after transplantation (12.60 kb, 95% CI: 11.52–13.70; *p* = 0.0005; Figure 3A). No significant difference was observed between one and two years after transplantation (*p* = 0.9841).

In contrast, recipients with recurrence of the underlying disease did not demonstrate significant changes in telomere length over time. Estimated telomere length was 9.23 kb (95% CI: 6.78–11.70) before transplantation, 11.79 kb (95% CI: 7.50–16.10) one year after transplantation, and 7.25 kb (95% CI: 2.96–11.50) two years after transplantation (all *p* > 0.05; Figure 3B).

These findings suggest that long-term telomere recovery following allo-HSCT is associated with the absence of disease recurrence, whereas no clear association was observed with the incidence of acute GvHD or CMV infection.

### 3.3. Distribution of TERT Variants in HSCT Recipients and Their Donors—Associations with Transplant Outcome

Patients and donors were genotyped for four various single nucleotide polymorphisms (SNPs), two located within the promoter region (rs2735940 and rs2853669) and two intronic substitutions (rs2736100 and rs10069690, in intron 2 and 4, respectively). No significant difference was observed between patients and donors carrying the same *TERT* genotype/allele. Genotype distributions for the analysed *TERT* polymorphisms in both recipients and donors are presented in Table 2.

All analysed polymorphisms were in Hardy–Weinberg equilibrium. Comparison of genotype and allele frequencies revealed no significant differences between recipients and donors. Furthermore, correlation analysis between individual genotypes and telomere length did not reveal any significant associations, indicating that none of the donor or recipient genotypes were associated with increases or decreases in measured PBMC telomere length.

Interestingly, some relationships were observed with the clinical transplant outcome.

**Figure 4:**
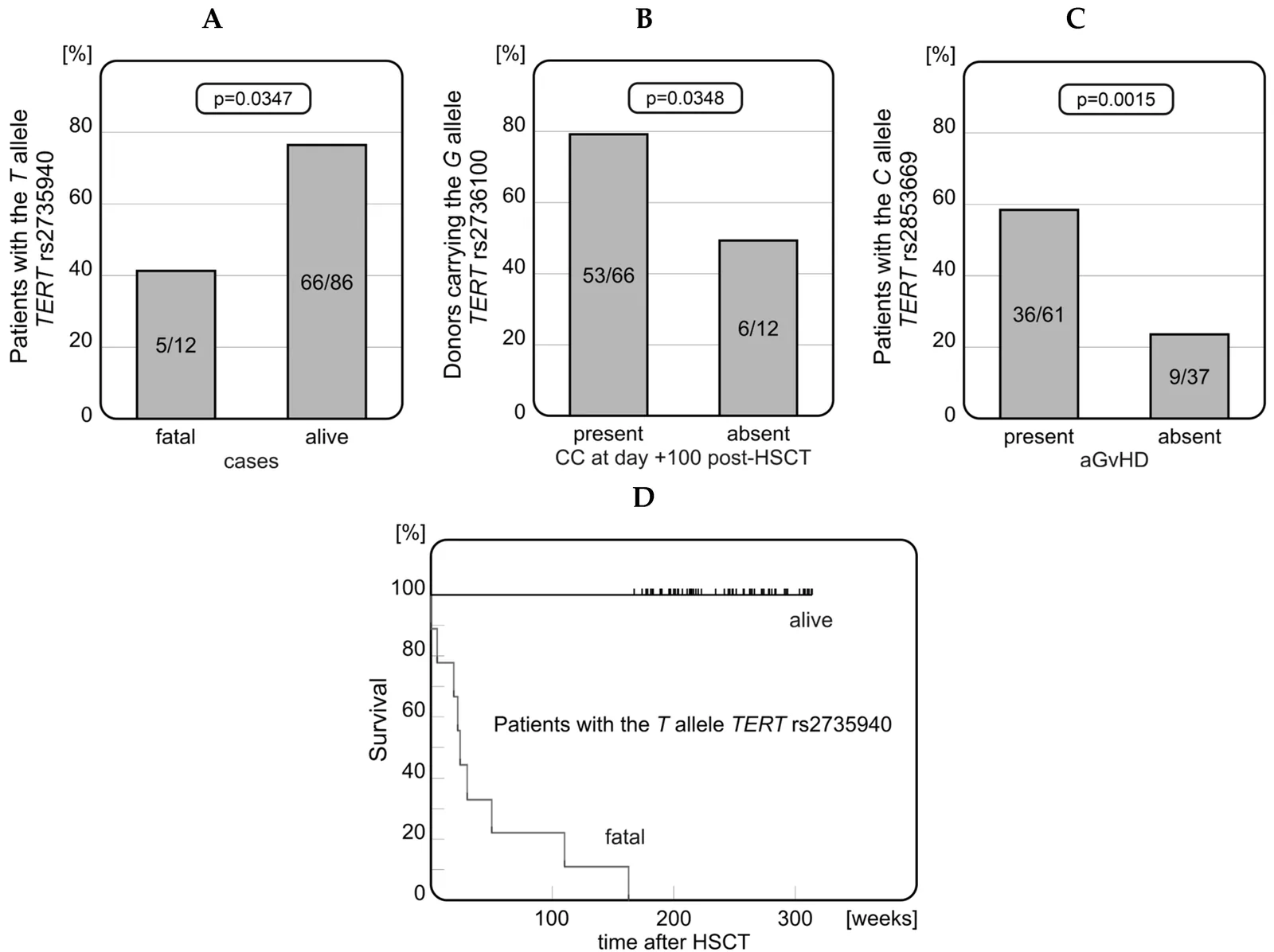
Associations of *TERT* gene polymorphism with transplantation outcome: (**A**) the *T* allele of *TERT*p rs2735940 with patient survival, (**B**) the *G* allele of donor *TERT*p rs2736100 with the achievement of complete chimerism (CC) at day 100 post-allo-HSCT in recipients, (**C**) the *C* allele of *TERT*p rs2853669 in patient groups after HSCT according to the occurrence of acute graft-versus-host disease (aGvHD). (**D**) Kaplan–Meier survival curve showing the overall survival of allo-HSCT recipients with the *T* allele of *TERT* rs2735940.

The occurrence of the *T* allele of the *TERT*p rs2735940 polymorphism differed between patients who died and those who survived after allo-HSCT. Chi-square analysis revealed a statistically significant difference between the groups (*p* = 0.0347; Figure 4A). The *T* allele was significantly more frequent—over four times higher—in patients who survived compared with those who died (OR = 4.62; 95% CI: 1.32–16.16; Figure 4A). Consistent with the observed association between the *T* allele of *TERT*p rs2735940 and survival status (Figure 4A), Kaplan–Meier analysis illustrates the difference in overall survival between patients classified as alive and those who died following allo-HSCT (Figure 4D). Moreover, the *G* allele of donor *TERT* rs2736100 (intron 2) was found to affect patient clinical outcomes regarding the achievement of complete chimerism (CC) at day 100 post-transplant. Analysis revealed a statistically significant difference between groups (*p* = 0.0348; Figure 4B). The *G* allele was significantly more frequent among donors of patients who achieved CC compared with donors of individuals who still exhibited mixed chimerism at day 100 (OR = 4.08; [95% CI]: 1.13–14.72; Figure 4B). Furthermore, the presence of the *C* allele of *TERT*p rs2853669 was significantly more frequent in children with aGvHD compared with patients without the complication (OR = 4.48; 95% CI: 1.81–11.11; *p* = 0.0015; Figure 4C).

### 3.4. Haplotype Analysis

**Figure 5:**
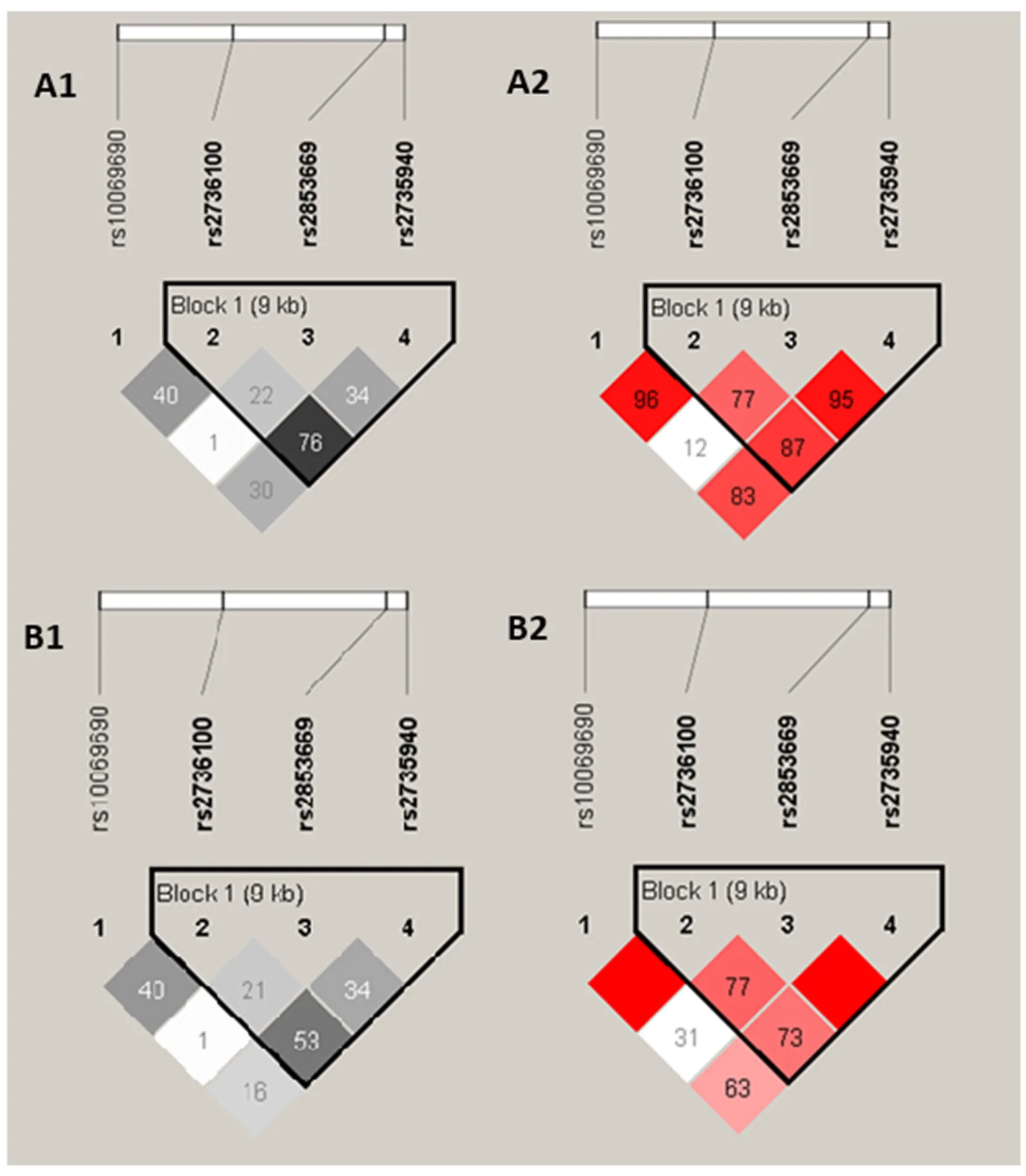
Linkage disequilibrium (LD) analysis of four *TERT* gene polymorphisms (rs10069690, rs2736100, rs2853669, rs2735940). (**A**) Recipients: (**A1**) r^2^ values, (**A2**) D′ values. (**B**) Donors: (**B1**) r^2^ values, (**B2**) D′ values. Red squares represent D′ values (strength of linkage), while shades of gray indicate r^2^ values (allele correlation).

Linkage disequilibrium (LD) analysis of four *TERT* polymorphisms (rs10069690, rs2736100, rs2853669, and rs2735940) revealed the presence of a haplotype block comprising three SNPs: rs2736100, rs2853669, and rs2735940, spanning approximately 9 kb (Figure 5). The strongest LD was observed between rs2853669 and rs2735940, with D′ values of 0.73 in donors and 0.76 in recipients. D′ values for the remaining SNP pairs ranged from 0.31 to 0.77 in donors and from 0.12 to 0.87 in recipients.

Analysis of r^2^ indicated the highest allele correlation between rs2853669 and rs2735940 (r^2^ = 0.53 in donors and r^2^ = 0.76 in recipients). These results suggest that the analysed variants are partially co-inherited, with stronger linkage observed in recipients compared with donors.

**Figure 6:**
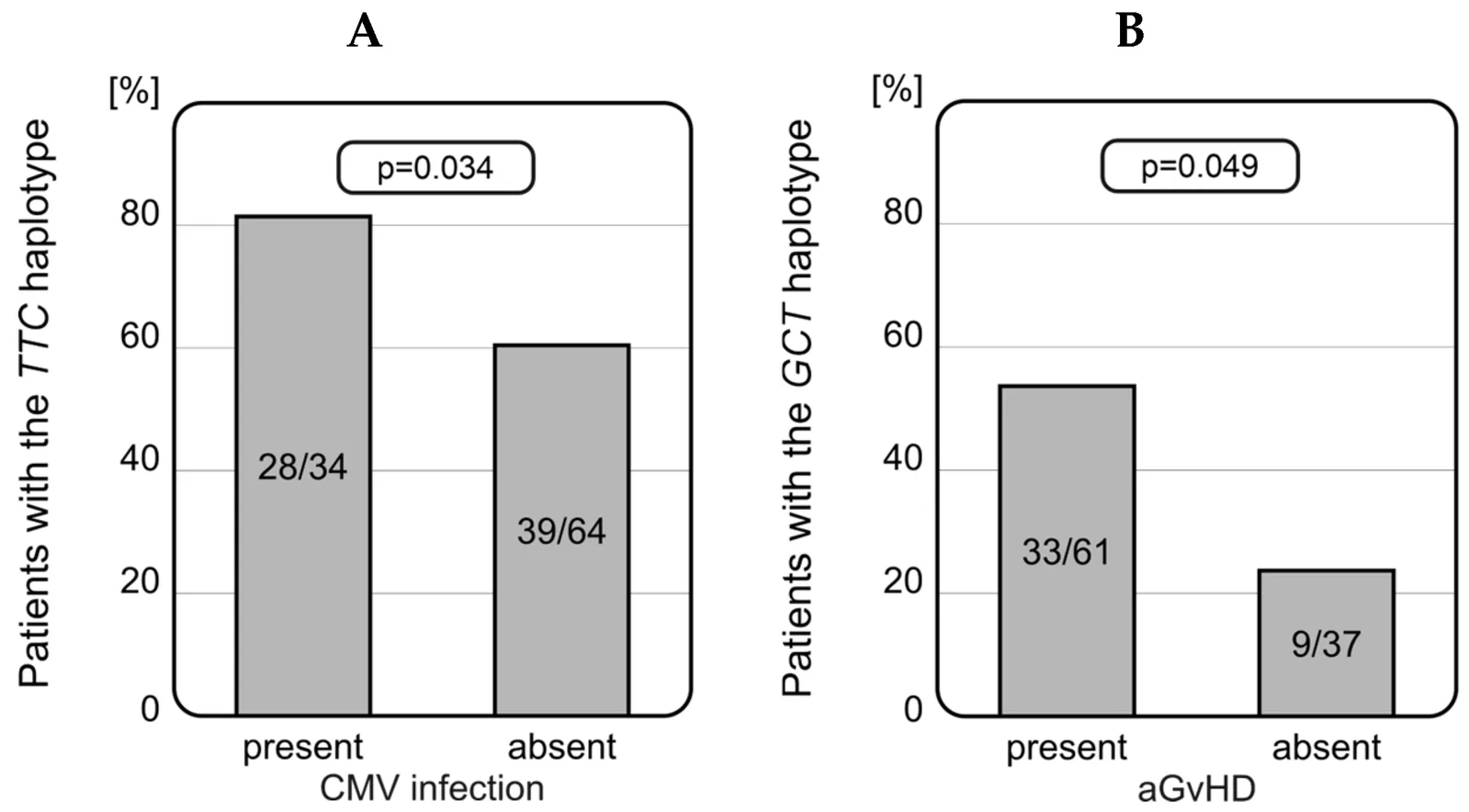
*TERT* rs2736100, rs2853669, rs2735940 haplotypes and transplantation outcome: (**A**) Association of the *TTC* haplotype of the *TERT* gene with the risk of cytomegalovirus (CMV) reactivation after allo-HSCT in recipients. (**B**) Relationship between the *GCT* haplotype of the *TERT* gene and the occurrence of acute graft-versus-host disease (aGvHD) in recipients.

Within the haplotype block comprising three SNPs (rs2736100 [intron 2], rs2853669 [*TERT*p], and rs2735940 [*TERT*p]), six major haplotypes were identified. The most frequent haplotype in both donors and recipients was *TTC* (*T* allele—rs2736100; *T*—rs2853669; *C*—rs2735940), with frequencies of 45.5% in donors and 43.8% in recipients. The next most common haplotypes were *GCT* (20.5% in donors, 26.5% in recipients) and *GTT* (20.5% in donors, 23.0% in recipients). Rare haplotypes included *GTC*, *TTT*, and *TCT* (≤7%). In the group of recipients, a significant association was observed between the TTC haplotype and the occurrence of CMV reactivation after allo-HSCT. CMV reactivation was seen in 41.8% of *TTC* haplotype carriers compared with 19.4% in the non-carrier group (χ^2^ = 4.71). Risk analysis indicated that the presence of the *TTC* haplotype was associated with an almost threefold increased likelihood of viral reactivation (OR = 2.99; [95% CI]: 1.08–8.25; *p* = 0.034; Figure 6A). This result remained borderline significant after applying Yates’ correction (*p* = 0.052). However, the *GCT* haplotype was significantly less frequent in recipients who experienced CMV reactivation after allo-HSCT (19.0%) compared with patients without reactivation (81.0%) (χ^2^ = 7.94). The presence of the *GCT* haplotype was associated with reduced risk of CMV reactivation (OR = 0.26; confidence interval [95% CI]: 0.10–0.65; *p* = 0.0041). In the recipient group, the *GCT* haplotype was significantly associated with a higher incidence of aGvHD. aGvHD was observed in 78.6% of children carrying the *GCT* haplotype compared with 50.0% of individuals without this allele combination (χ^2^ = 8.34; OR = 3.67; 95% CI: 1.48–9.06; *p* = 0.0049; Figure 6B).

## 4. Discussion

Comparison of telomere length before transplantation between paediatric patients and donors whose age did not exceed 18 years revealed significantly longer telomeres in the donors. This observation confirms the hypothesis that donor age is an important factor determining the length of hematopoietic cell telomeres. This result is consistent with reports by Akiyama et al. (1998) and Lai et al. (2022), which showed that younger donor age correlates with longer telomeres and better proliferative potential of transplanted cells in recipients [31,32]. In clinical practice, these findings may mean that a younger donor is an optimal source of hematopoietic cells, enabling faster and more effective reconstitution of the hematopoietic system and, thus, potentially better transplant outcomes and a lower risk of complications. In the context of this study, this finding may explain the observed telomere lengthening in recipients in the first year after transplantation. In the study by Dratwa-Kuzmin et al. (2025), adult patients were divided according to the median age at the time of allo-HSCT, which was 49 years [14]. It was noted that younger patients tended to have longer telomeres on average than older patients. These results, combined with the observations of this study, suggest that younger age—both of the donor and the recipient—may promote the maintenance of longer telomeres and higher proliferative potential of hematopoietic cells after allo-HSCT.

In this study, we observed differences in pre-transplant telomere length among patient groups receiving various conditioning regimens. Although recipients who underwent NMA conditioning had significantly longer telomeres than those who underwent MAC or RIC conditioning regimens, the intensity of conditioning was closely associated with the characteristics of the underlying disease in our cohort. In particular, the NMA group comprised predominantly patients with bone marrow failure disorders, whereas the MAC group consisted mainly of patients with ALL. Because the choice of conditioning regimen is determined, at least in part, by disease type and patient characteristics, the observed association between conditioning intensity and telomere length may therefore be influenced by confounding related to the underlying disease. Importantly, several of the disorders represented in the NMA group, particularly bone marrow failure syndromes, may themselves be associated with altered telomere biology. Thus, the longer telomeres observed in the NMA group should not be interpreted as evidence that less intensive conditioning directly preserves or is associated with longer telomeres. Rather, the findings may reflect differences in the clinical and biological characteristics of the patients selected for different conditioning strategies. These observations highlight the importance of considering underlying disease and other patient-related factors when evaluating associations between conditioning intensity and telomere length. Larger studies with more balanced disease distributions and multivariable analyses will be required to determine whether conditioning intensity independently contributes to variation in telomere length.

The age of the recipient and donor appears to be an important factor determining the initial telomere length and regenerative potential of hematopoietic cells after HSCT. However, this is not the only factor that may affect the dynamics of changes observed in this study. Another important aspect is the underlying disease that is the indication for transplantation, which may affect telomere length to varying degrees even before HSCT, both through the disease process itself and through previous treatments (e.g., chemotherapy, radiotherapy, and immunosuppression). These differences may partly explain the inconsistent results observed between different groups of patients. In the analysis of individual disease entities, only in the case of ALL patients was a statistically significant difference in telomere length observed in the context of the time points at which the observation was conducted. This result can be biologically justified by the specificity of the disease itself, which is characterized by extremely intense clonal proliferation and high telomerase activity. Karow et al. (2021) observed that telomeres in lymphoblast cells in a patient with ALL are significantly shorter compared to their length identified in B and T lymphocytes, which reflects exceptionally numerous clonal divisions and indicates high telomerase activity [33]. Engelhardt et al. (1998) described that in acute leukaemia’s in children (including ALL and AML), telomerase was intensely activated, which correlated with longer telomeres in peripheral blood compared to adult donors, although the mechanism of reconstruction after treatment differs between leukaemia’s [34]. Furthermore, Vakonaki et al. (2022) reported that the mean telomere length was significantly greater in remission samples (4.35 ± 1.75 kb) than in lymphoblasts collected at diagnosis (1.97 ± 0.90 kb) in patients with acute lymphoblastic leukaemia [35]. These findings suggest that telomere length increases following remission, likely reflecting the replacement of leukaemic blasts by normal haematopoietic cells after successful treatment [35]. From a biological perspective, it can be assumed that telomere length and polymorphic variability in the *TERT* gene modulate the proliferation and survival of T lymphocytes, which could potentially affect both the dynamics of the antiviral response and the development of alloimmune reactions. However, in clinical settings, this effect may be modified by a number of other factors, such as conditioning intensity, type of immunosuppression, or individual differences in the recipient’s immune response. The results indicate that telomere length is not an independent risk marker for major complications identified after allo-HSCT, but its role in more complex biological interactions is likely. Further studies on larger cohorts, taking into account both genetic and functional aspects, are necessary to fully assess the significance of telomeres in transplant success and the development of complications.

The study showed significant changes in the average telomere length in children who underwent allo-HSCT one year and two years after transplantation. In the group of paediatric recipients, an increase in telomere length was observed one year after HSCT, which may reflect thymus-dependent intensive immune renewal. The results also indicate an increase in the measured telomere length in the recipient’s PBMCs in the longer term following transplantation;, however, after one year, these telomeres are shorter, which is consistent with data showing that, following an initial phase of regeneration, telomeres stabilise and naturally shorten as a result of cell division. Furthermore, these results support the hypothesis that replacing the recipient’s haematopoiesis with donor-derived cells may contribute to changes in telomere length in PBMCs observed over time. In our cohort, donor chimerism was assessed at days +30, +60 and +90 after allo-HSCT, but these data were not available at the one- and two-year time points at which telomere length was analysed. We cannot therefore directly rule out the effect of changes in cellular composition at later time points.

Another important consideration is the cellular composition of the PBMC compartment, which undergoes substantial changes during immune reconstitution. The relative proportions of T cells, B cells, NK cells, and monocytes may differ between the recipient before transplantation, the donor sample, and the recipient during post-transplant immune reconstitution. Because these cell populations may have distinct telomere lengths, changes in their relative proportions could influence the mean telomere length measured in bulk PBMCs. We therefore cannot exclude the possibility that changes in cellular composition contributed to the longitudinal changes observed at later time points. Accordingly, the observed increase in mean telomere length in PBMCs should not necessarily be interpreted as telomere elongation within individual cells. Rather, it may partly reflect changes in donor-derived haematopoietic composition and immune-cell reconstitution over time.

Most studies [31,32,36,37] describe rapid telomere shortening after allo-HSCT, especially in the first months, which is logical in the context of intensive proliferation. However, these differences can be explained by several factors, including the young age of patients and donors—the younger the donor, the longer their telomeres and the higher the proliferative potential of the harvested cells. The results obtained are consistent with the available data in the literature. Children receiving cells from donors under 18 years of age had longer telomeres [31], and the age of the donor and the condition of their telomeres strongly correlate with the length of telomeres in recipients [32]. Furthermore, longer telomeres in the donor are associated with a better survival rate of the transplantation procedure [38,39]. In addition, higher telomerase activity in the hematopoietic cells of children may promote the restoration or even lengthening of telomeres in the first phase of hematopoietic reconstitution [40].

Furthermore, this study showed that specific polymorphic variants within the *TERT* gene were significantly associated with the course of hematopoietic reconstitution and the occurrence of specific complications after allo-HSCT. The *T* rs2735940 *TERT*p allele was more common in patients who survived after transplantation, which may suggest its potential protective effect. The presence of the *G* rs2736100 (intron 2) *TERT* allele in the donor correlated with a higher frequency of achieving complete chimerism (CC) on day 100 after allo-HSCT, indicating a possible role for this polymorphism in promoting successful engraftment of donor cells. In contrast, the *C* rs2853669 *TERT*p allele was significantly more frequently identified in patients who developed aGvHD, suggesting that this genetic variant influences the regulation of the immune response after transplantation. The relationships obtained are consistent with a growing number of reports indicating that specific variants of the *TERT* gene modulate telomere length and post-transplant course. Multicenter association analyses performed for rs2736100 *TERT* showed that alleles at this locus are associated with longer leukocyte telomere length (e.g., +0.026 T/S for the *C* allele), which confirms the potential influence of *TERT* on the telomere phenotype and may explain the relationships observed in the present study regarding the rate of achieving full engraftment [41]. Notably, the association between the rs2736100 variant in the *TERT* gene and haematological diseases is consistent with the concept that genetically determined differences in telomere length may influence disease phenotype and clinical course. Similar associations have been reported in studies of cancer susceptibility, including lung cancer and myeloproliferative neoplasms, where alleles associated with longer telomeres conferred distinct risk profiles. These findings further support the functional relevance of this locus and highlight the context-dependent effects of telomere biology [42,43]. Taken together, these data support the interpretation that the biology of donor and recipient telomeres (combining knowledge of the average telomere length with SNPs in *TERT*) co-determines the rate of hematopoietic recovery and the risk of complications after allo-HSCT. This is in line with our observation regarding the association of the rs2736100 *TERT* variant with the frequency of achieving CC and the association of *TERT*p rs2853669 with aGvHD. However, the differences in the direction of the effect reported in the literature suggest heterogeneity depending on the population, design, and endpoints.

In the haplotype analysis performed in allo-HSCT recipients, it was shown that specific haplotype variants of the *TERT* gene are significantly associated with the course of infectious and immunological complications. The most common *TTC* haplotype was found to be a risk factor for CMV reactivation. This result suggests that the *TTC* variant may modulate susceptibility to viral replication after transplantation, although the confidence interval is wide and requires confirmation in a larger cohort. An intriguing and seemingly contradictory result was the association of the *GCT* haplotype with both the risk of aGvHD and CMV reactivation. The *GCT* haplotype was significantly more common in patients with aGvHD (OR = 3.67), indicating its potential role in regulating the immune response. At the same time, *GCT* carriers were significantly less likely to experience CMV reactivation (OR = 0.26). This may suggest that this haplotype promotes stronger activation of the immune system, increasing the risk of GvHD but at the same time improving control over virus replication. These relationships are consistent with the concept that polymorphic variants can affect the proliferation and functionality of T lymphocytes. It should not be forgotten that telomerase plays a key role in prolonging the life of immune system cells, and genetic variability within the components (proteins) that make up this enzyme can modulate the dynamics of antiviral and alloreactive responses. The results obtained indicate that telomere length analysed after allo-HSCT in children is subject to dynamic changes and can only be explained to a small extent by the assessed clinical parameters. The literature emphasizes the role of recipient and donor age in maintaining regenerative potential, as well as the importance of *TERT* gene polymorphisms and haplotypes, which affect the rate of hematopoietic recovery and the risk of immune and infectious complications.

Several limitations of this study should be noted. Firstly, given the relatively small number of patients in some subgroups, statistically significant associations should be interpreted with caution. These observations need to be confirmed in larger, independent cohorts. A further limitation is that telomere length was assessed in whole blood samples rather than in specific cellular subpopulations. As telomere length may vary across different leukocyte subpopulations, whole-blood measurements provide a general estimate that may be influenced by differences in the relative proportions of circulating cellular populations. Consequently, these measurements may not fully reflect cell-specific differences in telomere length. Future studies incorporating analyses of specific cell subpopulations may provide a more detailed understanding of telomere dynamics and their potential association with genetic and clinical factors. Furthermore, the pre-transplant disease burden has not been uniformly characterised at the MRD level. Although most patients with malignancies were sampled in complete remission, residual disease and the intensity of prior antineoplastic therapy may influence baseline telomere length, and their contribution cannot be fully excluded. Despite these limitations, the present findings provide potentially significant observations that may serve as a basis for further research and validation within the framework of larger and more detailed analyses.

The results obtained did not clearly confirm these relationships, but suggest that telomere length assessment in combination with genetic analysis of the telomerase catalytic subunit (*TERT*) may in the future provide valuable clinical information and form the basis for further research in large paediatric cohorts.

## Figures and Tables

**Table 1:** Clinical characteristics.

Patients’ age (years)	
Median (Range)	7 (<1–21)
Donors age (years)	
Median (Range)	26 (3–57)
Patients’ Gender	
Male/Female	59/41
Source of transplant material	
Bone marrow (BM)	17
Peripheral blood stem cells (PBSC)	83
Type of Donor	
Matched unrelated donor (MUD)	78
Matched sibling donor (MSD)	19
Haploidentical	3
Complications after HSCT	
aGvHD I-IV/II-IV	62/29
Reactivation of viral infection	
Cytomegalovirus (CMV)	27
Epstein-Barr virus (EBV)	16
Diagnosis	
Acute lymphoblastic leukaemia	39
Acute myeloid leukaemia	9
Severe aplastic anaemia	9
Myelodysplastic syndromes	6
Wiskott-Aldrich syndrome	5
Chronic myeloid leukaemia	4
Juvenile myelomonocytic leukaemia	4
Paroxysmal nocturnal hemoglobinuria	3
SCID syndrome	3
Fanconi anaemia	2
Acute mixed-phenotype leukaemia	2
Hunter syndrome	2
X-linked lymphoproliferative syndrome	2
Other	10

**Table 2:** Genotype distributions for four analysed *TERT* polymorphisms in both recipients and donors.

	Recipients (*n* = 98)	Donors (*n* = 98)
**rs2735940 (*TERT*p)**		
*CC*	25 (25.50%)	27 (27.5%)
*CT*	45 (45.90%)	48 (49.0%)
*TT*	28 (28.60%)	23 (23.50%)
**rs10069690 (intron 4)**		
*AA*	9 (9.20%)	6 (6.10%)
*AG*	43 (43.90%)	41 (41.80%)
*GG*	46 (46.90%)	51 (52.10%)
**rs2736100 (intron 2)**		
*GG*	28 (28.60%)	21 (21.40%)
*GT*	45 (45.90%)	51 (52.10%)
*TT*	25 (25.50%)	26 (26.50%)
**rs2853669 (*TERT*p)**		
*CC*	10 (10.20%)	5 (5.10%)
*CT*	35 (35.70%)	36 (36.70%)
*TT*	53 (54.10%)	57 (58.02%)

## Data Availability

The data are stored in a password-protected repository and will be available at: https://cloud.hirszfeld.pl/index.php/f/1665605; (accessed on 14 July 2026) access will be granted by the corresponding author upon reasonable request.

## Abbreviations

The following abbreviations are used in this manuscript:

aGvHD	Acute Graft versus Host Disease
ALL	Acute Lymphoblastic Leukaemia
Allo-HSCT	Allogeneic Hematopoietic Stem Cell Transplantation
BM	Bone Marrow
CC	Complete Chimerism
cGvHD	Chronic Graft versus Host Disease
CMV	Cytomegalovirus
EBV	Epstein–Barr Virus
GvHD	Graft versus Host Disease
HLA	Human Leukocyte Antigen
HSCT	Hematopoietic Stem Cell Transplantation
LD	Linkage Disequilibrium
MAC	Myeloablative Conditioning
MC	Mixed Chimerism
MUD	Matched Unrelated Donor
NMA	Non-Myeloablative Conditioning
PBSC	Peripheral Blood Stem Cells
RIC	Reduced Intensity Conditioning
SNP	Single Nucleotide Polymorphism
TERT	Telomerase Reverse Transcriptase
